# Jungle yellow fever, Rio de Janeiro.

**DOI:** 10.3201/eid0703.010331

**Published:** 2001

**Authors:** A M Filippis, H G Schatzmayr, C Nicolai, M Baran, M P Miagostovich, P C Sequeira, R M Nogueira


Thai isolates. Only 20 different spoligopatterns were found among 499 isolates in the Vietnam study, compared to 60 among 244 isolates in our study.

Although the Beijing genotype of *M. tuberculosis* has been recognized in settings of emerging drug resistance around the world, the situation in Southeast Asian countries with a high frequency of Beijing type isolates appears to be nonuniform.

**Wolfgang M. Prodinger,* Porntip Bunyaratvej,† Roongnapa Prachaktam,† and Marion Pavlic***

*University of Innsbruck, Innsbruck, Austria; and †Faculty of Medicine, Ramathibodi Hospital, Mahidol University, Bangkok, Thailand

### References

1. Anh DD, Borgdorff MW, Van LN, Lan NTN, van Gorkom T, Kremer K, et al. *Mycobacterium tuberculosis* genotype Beijing emerging in Vietnam. Emerg Infect Dis 2000;6:302-5.
2. Kamerbeek J, Schouls L, Kolk A, van Agterveld M, van Soolingen D, Kuijper S, et al. Simultaneous detection and strain differentiation of *Mycobacterium tuberculosis* for diagnosis and epidemiology. J Clin Microbiol 1997;35:907-14.
3. Dale JW, Nor RM, Ramayah S, Tang TH, Zainuddin ZF. Molecular epidemiology of tuberculosis in Malaysia. J Clin Microbiol 1999;37:1265-8.
4. Niemann S, Richter E, Rusch-Gerdes S, Thielen H, Heykes-Uden H. Outbreak of rifampin and streptomycin-resistant tuberculosis among homeless in Germany. Int J Tuberc Lung Dis 1999;3:1146-7.
5. van Soolingen D, Qian L, de Haas PE, Douglas JT, Traore H, Portaels F, et al. Predominance of a single genotype of *Mycobacterium tuberculosis* in countries of east Asia. J Clin Microbiol 1995;33:3234-8.
6. Palittapongarnpim P, Luangsook P, Tansuphaswadikul S, Chuchottaworn C, Prachaktam R, Sathapatayavongs B. Restriction fragment length polymorphism study of *Mycobacterium tuberculosis* in Thailand using IS*6110* as probe. Int J Tuberc Lung Dis 1997;1:370-6.
7. Soini H, Pan X, Amin A, Graviss EA, Siddiqui A, Musser JM. Characterization of *Mycobacterium tuberculosis* isolates from patients in Houston, Texas, by spoligotyping. J Clin Microbiol 2000;38:669-76.

### Jungle Yellow Fever, Rio de Janeiro

**To the Editor:** Yellow fever control in Brazil through vaccination campaigns began in 1937. However, cases of jungle yellow fever still occur despite the existence of a potent vaccine and immunization campaigns focused on areas endemic for the jungle form of the disease (1). Most of these cases are in men in rural areas.

In Brazil from 1980 to 1998, 376 cases of jungle yellow fever were laboratory confirmed (by virus isolation, with or without immunoglobulin [Ig]M-capture enzyme-linked immunosorbent assay [MAC-ELISA] and immunoperoxidase stain), with 216 deaths (case-fatality rate 57.4%). Most cases were from Maranhão and Goiás States, with 99 and 41 cases, respectively; Goiás, in midwestern Brazil, reported a case-fatality rate of 95%.

During 1998 to 1999, 106 cases of jungle yellow fever were confirmed, with 40 deaths (37.7%). During 1999, 75 cases were confirmed, compared with 34 cases in 1998 and a mean of 20 cases per year from 1980 to 1998 (2). In 2000, 84 cases were confirmed, with 40 deaths (47.6% case-fatality rate). During 2000, the probable site of infection for nearly all cases was in Goiás, with 53 confirmed cases and 23 deaths, suggesting epizootic circulation of the virus (2). These cases were in unvaccinated persons who became ill in their home states after traveling to endemic areas for tourism or work.

In Brazil, almost two thirds of the territory is considered an enzootic area (3). Rio de Janeiro State is not endemic for jungle yellow fever, but in January 2000, the Oswaldo Cruz Institute confirmed a case of yellow fever in a 24-year-old woman who had traveled to a national park in Goiás State on December 28, 1999, with a group of 17 persons. Yellow fever infections were also confirmed in tourists from other states who visited this park in late 1999.

The young woman became ill on January 3 with fever, headache, retroocular pain, prostration, anorexia, and nausea. She returned to Rio de Janeiro on January 5 and visited a private clinic on January 7, when a complete blood count, platelet count, urea, creatinine, liver function tests, and dengue serologic testing were performed. The patient had leukopenia (1,730 leukocytes/$mm^3$), 100,000 platelets/$mm^3$, AST 911 U/L and ALT 680 U/L, creatinine 0.90 mg/dL, urea 10 mg/dL, and normal bilirubin and protein. Anti-dengue IgM serology was negative. A blood sample was collected January 11 for yellow fever diagnosis. Reverse transcription-polymerase chain reaction (RT-PCR) test was performed on RNA extracted from the serum (4), and virus isolation was attempted on C6/36 cells, both with negative results. A MAC-ELISA test was positive for yellow fever, with a serum IgM titer 1/80,000 8 days after onset of symptoms. The patient recovered within a week. After confirmation of this case in the only person who became ill in the travel group, yellow fever IgM serologic testing was performed on the other group members, all of whom tested negative. RT-PCR and virus isolation were not attempted because the sera were taken after the viremia period.

Control measures for the *Aedes aegypti* vector were promptly taken for a radius of 300 m around the patient's home. A vaccination campaign was carried out, during which 735 neighbors were vaccinated. An epidemiologic survey was conducted in the area by using active surveillance for all symptomatic cases of fever during the period of yellow fever transmissibility. Blood samples from patients with fever were assessed for yellow fever virus and antibodies. Surveillance was intensified immediately in Rio de Janeiro State, and our laboratory examined 54 sera from patients who had traveled recently to endemic areas and who had compatible signs and symptoms (in accordance with a nationwide protocol). All these persons tested negative for yellow fever.

From January to July 2000, >16.9 million people were vaccinated against yellow fever (2); however, cases continue to occur. Unvaccinated persons who visit yellow fever-endemic areas pose a high risk of introducing jungle yellow fever cases into nonendemic areas.

**Ana M.B. Filippis,* Hermann G. Schatzmayr,* Cecília Nicolai,† Mary Baran,† Marize P. Miagostovich,* Patrícia C. Sequeira,* and Rita M.R. Nogueira***

*Oswaldo Cruz Institute, Oswaldo Cruz Foundation-FIOCRUZ, Rio de Janeiro, Brazil; and †Municipal Health Department, Rio de Janeiro, Brazil

**References**

1. Barros MLB, Boecken G. Jungle yellow fever in central Amazon. Lancet 1996;348:969.
2. Ministry of Health. Notified cases of yellow fever by state, Brazil. Brasilia: National Epidemiology Center; 2000.
3. Vasconcelos PFC, Rodrigues SG, Degalier N, Moraes MAP, Rosa JFST, Rosa EST, et al. An epidemic of sylvatic yellow fever in the Southeast Region of Maranhão State, Brazil, 1993-1994: epidemiological and entomological findings. Am J Trop Med Hyg 1997;57:132-7.
4. Deubel V, Huerre M, Cathomas G, Drouet M-T, Wuscher N, Le Guenno B, et al. Molecular detection and characterization of yellow fever in blood and liver specimens of a non-vaccinated fatal human case. J Med Virol 1997;53:212-7.

### Emergence of Metronidazole-Resistant *Bacteroides fragilis*, India

**To the Editor:** Members of the *Bacteroides fragilis* group are the most commonly isolated anaerobic pathogens in humans. Metronidazole has been the drug of choice for preventing and treating such infections for 40 years. Although *B. fragilis* exhibits the broadest spectrum of recognized resistance to antimicrobial agents among anaerobes, the worldwide rate of metronidazole resistance remains low, <5% (1,2). We report here the first metronidazole-resistant strain of *B. fragilis* from India.

A 34-year-old man with myelodysplastic syndrome was admitted to our hospital with a short history of myalgia, general malaise, and bleeding gums. Bone marrow examination showed evidence of severe aplastic anaemia, for which he was treated with cyclophosphamide and blood transfusions. Ceftazidime and amikacin were also administered empirically for febrile neutropenia. The patient remained in the intensive care unit of our medical oncology ward and was given repeated courses of chemotherapy and blood transfusions. He also had repeated episodes of febrile neutropenia, which resolved with a combination of vancomycin, aminoglycosides, and third-generation cephalosporin. After 4 months in the hospital, during an episode of febrile neutropenia, the patient's condition started to deteriorate, and high-grade fever developed. Physical examination showed temperature of 38°C, heart rate 80/min, blood pressure 100/70 mmHg, and marked pallor. Laboratory investigations showed a hemoglobin level of 4g/dL and marked neutropenia (absolute neutrophil count 320/mm$^3$). Liver and renal function test results were within normal limits. Peripheral blood smears were negative for malarial parasites. Culture of urine revealed no growth, and the Widal test was negative. Two blood samples were collected in Wampole isolator tubes (Wampole Laboratories, Cranbury, NJ), for isolation of aerobic and anaerobic bacteria. Subsequently, intravenous antimicrobial therapy with vancomycin, metronidazole, and ceftazidime was started. The patient died a day after collection of blood for culture.

Antemortem blood cultures grew *Pseudomonas aeruginosa* and *B. fragilis.* The isolate of *B. fragilis* was identified by conventional tests and Rap ID ANA II system (Innovative Diagnostic System, Norcross, GA). *P. aeruginosa* was sensitive to piperacillin but resistant to amikacin, ceftazidime, cefotaxime, and ciprofloxacin. *B. fragilis* was resistant to metronidazole (MICs, 256 µg/mL) by both standard broth dilution method and E-test (AB Biodisk, Solne, Sweden). The isolate was also resistant to cefotaxime and ceftazidime. However, it was sensitive to chloramphenicol, clindamycin, and imipenem.

Primary bacteremia caused by anaerobic organisms accounts for <5% of septicemia in cancer patients (3). Chemotherapy is a known predisposing factor for anaerobic bacteremia because it causes gastrointestinal ulceration, which permits anaerobes to enter circulation (4).

Anaerobic bacteremia is usually polymicrobial in etiology and has a high death rate (4). In this case, both bacterial isolates were resistant to the empirical treatment. Delay in initiating appropriate therapy was perhaps a major contributor to the patient's death.

Metronidazole is the drug of choice for empirical coverage of anaerobic infections. The precise incidence of resistance to metronidazole in *B. fragilis* isolates is difficult to estimate (5), since routine antimicrobial sensitivity testing of anaerobes is not being done by most laboratories in the world. Published articles reveal only a few reported cases of *B. fragilis* that were resistant to metronidazole (6-10). Although the incidence of resistance to penicillin, cephalosporins, and clindamycin is increasing dramatically, no resistance to metronidazole in *B. fragilis* was found in some large-scale studies done throughout the world (11,12).

The true incidence of metronidazole resistance in India too is possibly underestimated since antimicrobial sensitivity testing is not being done routinely. However, we are conducting antimicrobial susceptibility testing of all anaerobic isolates in our institute. In a previous study we conducted (13), contrary to this report, none of 32 clinical isolates belonging to the family *Bacteroidaceae* obtained over a 5-year period were resistant to metronidazole.

Recently, the anaerobic reference unit in the UK noted a possible increase in the incidence of metronidazole resistance in *B. fragilis*, an observation that would have major implications for clinical microbiology laboratories, as well as for prophylactic and treatment regimens (5).

There is now a growing debate whether in vitro susceptibility testing should be performed for all *Bacteroides* isolates to guide antimicrobial therapy. The acquisition of metronidazole resistance by *B. fragilis* reported here from India emphasizes the need for a study to assess more accurately the susceptibilities of clinical isolates of *Bacteroides* spp.

Diagnostic microbiology laboratories and clinicians should be aware that the incidence of metronidazole resistance in clinically significant anaerobes may be increasing (5). Since antimicrobial resistance in anaerobes varies from one hospital to another and between different geographic locations, all hospitals should survey their sensitivity patterns and report any emerging resistance.

**Rama Chaudhry, Purva Mathur, Benu Dhawan, and Lalit Kumar**
All India Institute of Medical Sciences, New Delhi, India

**References**

1. Finegold SM, Wexler HM. Present status of therapy for anaerobic infections. Clin Infect Dis 1996;23(Suppl 1):S9-14.

